# Membrane Sensor Histidine Kinases: Insights from Structural, Ligand and Inhibitor Studies of Full-Length Proteins and Signalling Domains for Antibiotic Discovery

**DOI:** 10.3390/molecules26165110

**Published:** 2021-08-23

**Authors:** Pikyee Ma, Mary K. Phillips-Jones

**Affiliations:** 1 Laboratory of Biomolecular Research, Paul Scherrer Institute, CH-5232 Villigen, Switzerland; pik-yee.ma@psi.ch; 2 National Centre for Macromolecular Hydrodynamics, School of Biosciences, University of Nottingham, Sutton Bonington LE12 5RD, UK

**Keywords:** antibiotic discovery, two-component signal transduction systems, histidine kinase, membrane protein, ligand binding, inhibitor screening, transmembrane domain, vancomycin resistance, quorum sensing, nanodiscs, detergent

## Abstract

There is an urgent need to find new antibacterial agents to combat bacterial infections, including agents that inhibit novel, hitherto unexploited targets in bacterial cells. Amongst novel targets are two-component signal transduction systems (TCSs) which are the main mechanism by which bacteria sense and respond to environmental changes. TCSs typically comprise a membrane-embedded sensory protein (the sensor histidine kinase, SHK) and a partner response regulator protein. Amongst promising targets within SHKs are those involved in environmental signal detection (useful for targeting specific SHKs) and the common themes of signal transmission across the membrane and propagation to catalytic domains (for targeting multiple SHKs). However, the nature of environmental signals for the vast majority of SHKs is still lacking, and there is a paucity of structural information based on full-length membrane-bound SHKs with and without ligand. Reasons for this lack of knowledge lie in the technical challenges associated with investigations of these relatively hydrophobic membrane proteins and the inherent flexibility of these multidomain proteins that reduces the chances of successful crystallisation for structural determination by X-ray crystallography. However, in recent years there has been an explosion of information published on (a) methodology for producing active forms of full-length detergent-, liposome- and nanodisc-solubilised membrane SHKs and their use in structural studies and identification of signalling ligands and inhibitors; and (b) mechanisms of signal sensing and transduction across the membrane obtained using sensory and transmembrane domains in isolation, which reveal some commonalities as well as unique features. Here we review the most recent advances in these areas and highlight those of potential use in future strategies for antibiotic discovery. This Review is part of a Special Issue entitled “Interactions of Bacterial Molecules with Their Ligands and Other Chemical Agents” edited by Mary K. Phillips-Jones.

## 1. Introduction

Sensor histidine kinases (SHKs) (also known as histidine protein kinases) are usually membrane-bound proteins that constitute an integral part of short signalling pathways in bacteria known as two-component signal transduction systems (TCSs). TCSs classically consist of two classes of paired regulatory proteins—the aforementioned SHK component which serves as a modulator or transmitter protein involved in sensing changes in a particular environmental stimulus or set of stimuli, and a partner response regulator (RR, effector protein) which effects an appropriate adaptive response, often a change in gene expression. Recognition of such pairing between two different regulatory protein classes was first made by Ausubel and his colleagues [1] and a review of the historical background to their relatively “late” discovery in the 1980 s is given by Magasanik (1995) [2].

### 1.1. General Scheme and Mechanism of Signal Transduction Amongst TCSs

**Figure 1:**
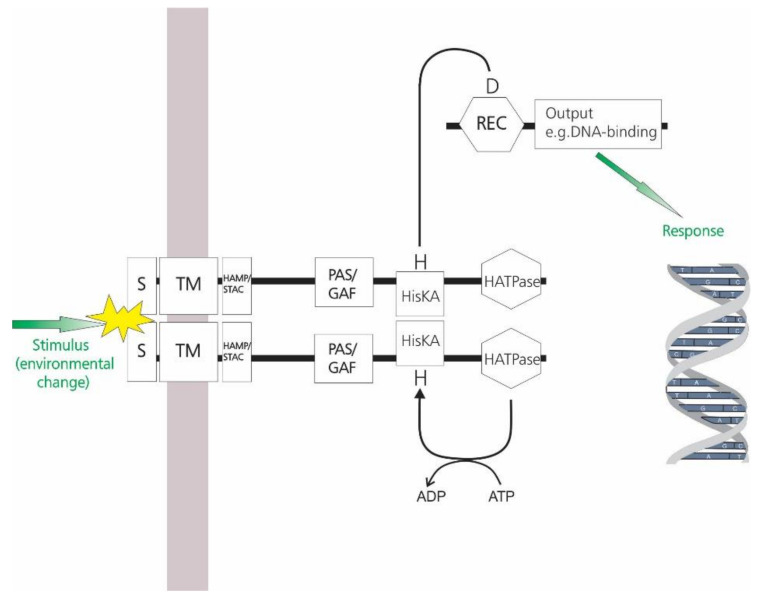
Schematic representation of a classic two-component signal transduction system (TCS). Changes in the levels of an environmental signal are perceived by the sensory domains of the sensor histidine kinase (SHK) component which is usually membrane-bound. Sensory domains may include periplasmic or extracellular domains (S). Cytoplasmic domains involved either in signal input or in signal transmission may also be present e.g., PAS or GAF domains, and/or HAMP or STAC signal transduction domains; helical linkers involved in signal transmission may also be present (not shown); HisKA, Histidine Kinase A (phosphoacceptor) (or DHp) domain; HATPase, catalytic domain. Together, the HisKA and HATPase domains constitute the “transmitter” or “kinase core” domain. Upon signal perception, the HisKA is activated, resulting in phosphorylation of the conserved Histidine (H) within the HisKA domain at the expense of intracellular ATP via the HATPase domain. SHKs generally function as dimers, with dimerization mediated by the HisKA domain to form a four-helix bundle. Autophosphorylation occurs either by trans-phosphorylation from the monomeric HATPase domain in one monomer to the Histidine residue within the HisKA domain of the second monomer (e.g., [3,4], or by *cis*-phosphorylation (each monomer phosphorylates itself) (e.g., [5,6]. Phosphotransfer occurs through interactions between the HisKA domain of the SHK and the receiver domain of the partner RR (top right). This results in phosphorylation at the conserved Aspartate residue of the RR which in turn affects the activity of the C-terminal “effector” domain (which may be a DNA-binding domain, for example) and thus bringing about an appropriate adaptive response to the original environmental stimulus.

In the classic scheme, the membrane SHK senses a change in a particular environmental, intramembranous or intracellular stimulus and in response becomes autophosphorylated (at the expense of intracellular ATP), at a conserved Histidine residue. The phosphoryl group is then transferred directly to the receiver domain (REC) of the partner RR at a conserved Aspartate residue usually resulting in a conformational change in the RR protein which results in altered affinity for a DNA-binding or other target site in the cell, or altered associated enzyme activities, thereby bringing about an appropriate adaptive response to the original stimulus (Figure 1 [3,4,5,6]). For recent Reviews of the detailed molecular mechanisms involved, see references [7,8,9,10,11,12,13]. 

### 1.2. Occurrence and Distribution of TCS Proteins amongst Prokaryotes Including Agents of Infection 

The distribution of TCSs is wide; they are found in members of the Bacteria, Archaea and Eukarya superkingdoms, but absent from mammals leading to the suggestion that they constitute good targets for the development of novel antibacterial drugs [14,15,16,17,18,19,20,21,22,23,24,25]. One post-genomic study reported the presence of TCSs in 864 out of 899 of the available fully sequenced genomes of the Bacteria, with all 21 phyla represented [20]. Indeed, ~50,000 TCS proteins have been identified [26] and the reader is referred to the SMART (Simple Modular Architecture Research Tool) web tool (http://smart.embl.de/) for updates on the identification and annotation of SHK and RR domains [27], and to other TCS-specific web resources including the P2CS (Prokaryotic Two-Component Systems, http://www.p2cs.org) [28], MiST2 (Microbial Signal Transduction, http://mistdb.com) [29], and the Census of Prokaryotic Response Regulators (http://www.ncbi.nlm.nih.gov/Complete_Genomes/RRcensus.html) [30] databases (all accessed on 16 September 2020) (see also [31]). The absence of TCSs generally occurs in parasitic species (such as *Mycoplasma* spp.) and endosymbionts (*Amoebophilus* spp.) that have significantly reduced genomes. Amongst the Archaea, TCSs are present in only approximately 50% of genomes, including 33 of the 42 genomes of the phylum *Euryarchaeota* and the one genome available for *Thaumarchaeota* [20]. 

Most bacterial pathogens including opportunistic, non-obligate parasitic pathogens possess multiple pairs of TCSs. For example, genome sequencing and other methods reveal 30 SHK and 34 RR proteins in *Escherichia coli* [32], whilst in *Enterococcus faecalis* there are 17 SHK and 18 RR proteins (17 pairs, and one orphan response regulator) [33,34]. The number of TCSs per species tends to be higher in environmental species that are exposed to fluctuations in the natural environment and that possess larger genomes. Some clinically-significant examples are presented in Table 1 [15,33,34,35,36,37,38,39,40,41,42,43,44,45,46,47,48,49,50,51,52,53,54,55,56].

TCSs are involved in a large range of important bacterial processes, including control and regulation of bacterial growth, biofilm formation and development, quorum sensing, coordinated virulence factor expression, sporulation, and antibiotic resistance determinants including drug efflux pumps (e.g., [14,17,23,25,57]). Their involvement in all these processes, coupled with their aforementioned absence in mammals and their distinct protein-histidine phosphorylation mechanism that contrasts with the serine/threonine and tyrosine phosphorylation of higher eukaryotes, has led to their consideration as possible antimicrobial targets for new antimicrobials. Furthermore, the strong structural homologies exhibited amongst the catalytic and receiver domains of SHKs and RRs, together with their multiplicity in a single bacterium offers opportunities to find single drugs that inhibit several crucial processes at once, thereby increasing the chances of lethality. It is also worth mentioning that a small number of TCSs have been shown to be essential in both Gram-positive and Gram-negative species, including three systems in *Helicobacter pylori* [58] and the WalRK TCS originally identified in *Bacillus subtilis* (YycGF) [59] which occurs in several Gram-positive pathogens of low guanosine-cytosine (GC) content belonging to the phylum of *Firmicutes*, including staphylococci, streptococci and enterococci [60]. This latter TCS (given different names in different species (YycFG, WalRK, VicRK)), has attracted particular attention with regard to novel chemicals agents that inhibit its activities, and although the essentiality of such TCSs often resides with the RR component, many inhibitor studies have focused on the membrane-located SHK components (see [61] for a recent listing of some examples). Several additional recent reviews of inhibitors of SHK and/or RR components have been published [17,18,21,23,25,62,63,64,65,66]. 

Despite much excellent progress, to the best of our knowledge, none of the inhibitors have yet been adopted in the clinic or indeed been included in clinical trials. There are a few reasons why this may be the case. Drug discovery programs thus far have probably been insufficiently wide with regard to the variety of potential inhibitors tried, and require use of more information derived specifically from TCSs from clinically-important pathogens [23]. In addition, the majority of studies have been conducted in academic laboratories, rather than by pharmaceutical industry, suggesting that with fewer resources within academic environments to push forward new leads there may yet be promising antibiotics waiting to emerge. Alternatively, it is also possible that toxicity studies were undertaken and candidate inhibitors failed at this stage. With regard to SHK-specific inhibitors, there is also a paucity of knowledge on how agonists and antagonists bind to some target sites within SHK proteins; increased knowledge in this area would be beneficial for structure-based drug design strategies, which are currently less popular but nonetheless worthy of continued pursuance. Whilst the structures and functions of many individual SHK domains, domain hybrids and multi-domain SHKs are known, including natively soluble SHKs that lack transmembrane segments [67,68,69,70,71,72], all of which have contributed significantly to knowledge of these proteins, there remains a lack of structural data on full-length membrane SHKs which results in part from the technical challenges associated with their purification as intact active membrane proteins in sufficient milligram quantities for elucidation of their three-dimensional structures by crystallisation or other methods [73]. However, these challenges are being overcome and in this Review, we describe some of the emerging successes in the overexpression and purification of full-length active membrane SHKs, the methods used for their solubilisation and reconstitution using detergents, amphipols, liposomes, and nanodiscs, and some of the consequent successes in structural and ligand binding studies, thereby expanding upon and complementing other Reviews of SHKs to date, which have focused less on studies of full-length purified proteins. Whilst use of intact membrane SHKs is useful for studies of ligand identification/binding (as we have pursued to date, e.g., [34,74,75]), and subsequent signal transduction events, they are equally applicable for inhibitor identification, which to date has been less actively pursued (though see [76]). Intact SHKs could potentially be used in primary screening for inhibitors of SHK enzyme activity, if for example they were incorporated within adapted versions of some previously described activity-based screening assays as described in [61,77,78] which currently employ domain fragments rather than intact SHKs. Alternatively, and probably more promisingly, they can serve as complementary tools to verify and further characterise initial inhibitor “hits” identified against SHKs and TCS signalling in general using whole cell or other reporter-based high-throughput screens (HTS) [63,79,80,81,82,83], phenotypic HTS targeting TCS-dependent responses [62,84,85], differential growth assays using a temperature-sensitive *yycF* mutant [16], high-throughput genetic systems for targeting homodimerization of SHKs [16], or structure-based virtual screening (SBVS) methods [86]. One advantage of including intact SHKs in drug discovery is being able to identify whether drug candidates bind and disrupt activities of the kinase specifically (rather than, for example, a linked RR in a reporter system), with possible inhibition sites blocking recognition of the in vivo signalling ligand, signal transmission across the membrane, propagation to catalytic domains, dimerization activities, or phosphotransfer or dephosphorylation activities. Another advantage is the contribution to knowledge made by understanding how signals are transmitted throughout the entire protein, including the conformational changes that occur. However, as mentioned above, SHKs in their full-length and intact (active) forms are far more technically challenging to produce than soluble versions, as they are predominantly membrane proteins and therefore more hydrophobic in nature. This aspect is not always fully appreciated and can be easily underestimated. Yet reliable and consistent methods do now exist for the overexpression, purification, and solubilisation of intact SHKs and the next section describes some of these methods and how the resulting purified proteins are prepared for investigation. 

## 2. Methodology for Producing Purified Active Forms of Full-Length Membrane SHKs

The strategy most commonly used for producing full-length SHKs heterologously is based on the expression of recombinant proteins using membrane protein expression plasmids such as pTTQ18His and *E. coli* as expression host, as first described in [87], a strategy adapted from that originally designed for the more hydrophobic bacterial membrane transporter and drug efflux protein families [88]. Expression from pTTQ18His is driven from an IPTG-inducible *tac* promoter which is controlled by LacI repression provided by the lacI^Q^ gene present on the plasmid. The advantage of this plasmid is that expression should be possible in almost any suitably engineered *E. coli* host independently of T7 polymerase-dependent systems. Use of pTTQ18His as expression plasmid has resulted in a high degree of success for producing intact SHKs compared with members of other membrane protein families, including successful expression of 15 of the 16 genome complement of membrane SHKs of *Enterococcus faecalis* [34], VanS (A-type) of *E. faecium* [75,89], BlpH, and ComD2 pheromone-sensing SHKs of *Streptococcus pneumoniae* (together with several other membrane proteins associated with antimicrobial resistance) [90] and the PrrB (or RegB) redox-sensing SHK protein of the Gram-negative bacterium *Rhodobacter sphaeroides* [87,91]. Other membrane overexpression plasmids include pT7-7 [92] based on T7 polymerase [93], pProEX available commercially [94] and pETCH [95] derived from SUMO fusion vectors [96]. Many studies have opted to express SHK genes as synthetic codon-optimised genes, though high success rates were obtained with plasmid pTTQ18His using the native gene sequences from both Gram-positive and Gram-negative species. The high rates of expression success obtained using all these plasmids may be attributable, at least in part, to the fewer α-helical membrane-spanning regions generally found amongst SHKs (2–7 transmembrane segments (TMs) [33]) compared with the more hydrophobic membrane transport and drug efflux proteins which can possess up to 10–14 TMs [88,97,98]. For further examples of membrane protein expression plasmids for use in bacterial hosts, the reader is referred to [88,99].

Following successful expression, yields of resulting recombinantly-expressed, purified proteins are usually low (0.1–3 mg/litre *E. coli* culture) compared to soluble proteins, necessitating large-scale cultures and optimisation of growth conditions (growth medium composition, culture temperature pre- and post-induction with inducer, time permitted post-induction prior to cell harvesting), detergent solubilisation conditions from *E. coli* membranes (detergent choice) and purification strategies, in order to optimise protein yields. Many of these considerations are described for hexa-Histidine tagged membrane transport and efflux proteins in the comprehensive review of [88]. However, there are some notable differences that apply to membrane SHKs: in our experience, higher rates of successful expression can be expected for SHKs compared with transporters and efflux proteins; and a relatively wide range of growth media can be used to cultivate *E. coli* hosts harbouring pTTQ18His-SHK constructs for successful expression outcomes [34,100]. These differences suggest that the SHK class of membrane protein is better tolerated by the *E. coli* host, perhaps because of the aforementioned lesser hydrophobicity of the proteins and/or because of the widespread nature of multiple membrane SHKs amongst bacteria including *E. coli* itself [32] (Table 1). This better tolerance is also reflected in rates of subsequent detergent-solubilisation and purification successes; for example, using pTTQ18His-based expression systems, 12 out of the 15 (80%) expressed membrane SHKs originating from *E. faecalis* were successfully purified from *E. coli* as active full-length (intact) proteins [34]. Table 2 summarises the high rates of success reported by Ma et al. (2008) at each individual step during the heterologous expression and purification process for the genome complement of 16 membrane SHKs of *E. faecalis* [34].

In our experience, instability amongst purified proteins has been infrequently encountered, provided purified samples are: (a) freshly prepared (with low temperature working) prior to their use in experiments; and (b) checked to confirm protein integrity post-purification and pre-experimentation. In the case of pTTQ18-based systems, the integrity of purified proteins can be conveniently confirmed not only by mass spectrometry but also through confirmation of the presence of the expected C- and N-terminal sequences, performed by Western blotting using a His tag antibody (which detects the C-terminal RGS-His tag sequence) and direct N-terminal sequencing, respectively. Additional precautions that could be tried (though were not usually necessary in our own studies) include use of protease inhibitors cocktails during protein preparation, introduction of thermostabilizing mutations by alanine scanning methods (as successfully employed for GPCRs) and identification and mutation of degradation sites (taking care to test the effects on function and structural integrity). 

In most examples described above, purification of full-length SHKs involved detergent solubilisation from membranes and employment of detergents in subsequent purification steps to maintain SHK solubility and stability. Alternatives to detergent have also been used, to generate conditions more closely resembling native membranes. For instance, following detergent solubilisation from host membranes, purified proteins can be reconstituted into phospholipid liposomes [101,102], detergents replaced with amphipols (amphipathic polymers that wrap around hydrophobic regions of membrane proteins to mask them from aqueous solvents) [103] or purified proteins inserted into nanodiscs (soluble nanoscale particles of lipid bilayer surrounded by an annulus of amphipathic protein —a membrane scaffold protein) [104]. Co-polymers such as styrene-maleic acid have been used to extract membrane proteins and lipids directly from host membranes to form soluble nanoparticles or native nanodiscs, thereby permitting purification of membrane proteins without their removal from a lipid environment [105,106]. Nanodisc technologies have been increasingly used in recent years for studies of bacterial membrane proteins, including SHKs [107,108,109], multi-drug efflux transporters [110,111], glycerol facilitators [112], beta-barrel outer membrane proteins [113,114], ABC transporters [115] and other bacterial membrane proteins (e.g., [116,117]). Cuozzo and Soutter (2014) reviewed how these newer technologies alongside more traditional detergent-based methods of producing membrane proteins impact on small-molecule screening for antibiotic discovery [118]. 

The following sections describe examples of the employment of full-length or sensory domain and/or TMD fragments to characterise structural features of SHKs, their mechanism of signal sensing and signal transduction across the membrane and beyond or features of ligand/inhibitor binding. Most use proteins in detergent micelles but studies employing other membrane protein solubilisation tools are highlighted alongside the discussions of each SHK study described below. 

## 3. Identification and Characterisation of Signalling Ligands and Inhibitors Using Full-Length SHKs

Ligands and inhibitors of SHKs can be identified and characterised by measuring changes in SHK activities and/or through conformational changes that occur due to ligand/inhibitor binding. Measurable activities include: (a) autophosphorylation—ligands usually increase autophosphorylation activity, but both activation and repression can be measured using purified intact SHKs because most exhibit measurable autophosphorylation activity even in the absence of ligand [34]; (b) phosphotransfer; and/or (c) phosphatase activities—the latter two both requiring purified RR as well as SHK in the assays (see ref. [87] for the methodology for all three assays). From the handful of signalling ligand and inhibitor studies undertaken so far using full-length SHKs, the majority have focused mainly on autophosphorylation activities. Ligand binding studies have employed a range of biophysical methods including CD, AUC, and fluorescence. Some recent examples of these approaches used to investigate the structural features of full-length SHKs, their activities and ligand/inhibitor binding are now described. 

### 3.1. Effect of Gelatinase Biosynthesis-Activating Pheromone (GBAP) on Autophosphorylation Activity of Intact FsrC 

**Figure 2:**
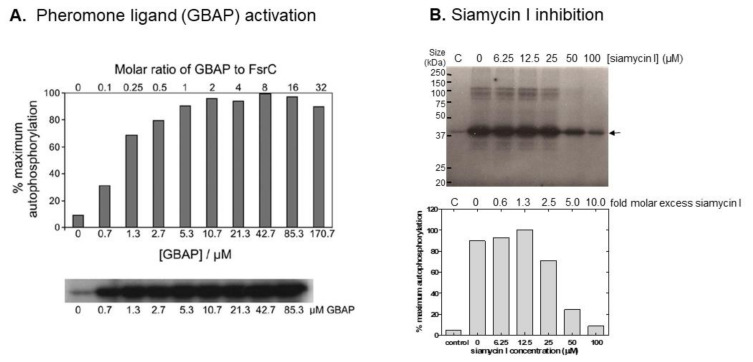
Activation (**A**) and inhibition (**B**) of autophosphorylation activity of purified detergent-solubilised intact FsrC. (**A**) Autophosphorylation reactions containing 80 pmoles FsrC pre-incubated for 20 min in the presence or absence of different GBAP concentrations, were initiated, terminated, and visualised as described previously [34]. Autoradiograph of phosphorylated FsrC proteins after 60 min incubation with different concentrations of GBAP are shown, together with quantitation of phosphorylated protein bands determined by phosphorimagery. (**B**) Inhibition of FsrC autophosphorylation activity by siamycin I. Autophosphorylation assays of FsrC (80 pmoles) were undertaken in the presence of a range of siamycin I concentrations as described in [76]. Upper panel: autoradiograph of phosphorylated FsrC proteins in the absence (C, control) and presence of 2-fold GBAP pheromone (10.7 µM); lower panel shows quantitation of phosphorylated protein bands determined by phosphorimager. Panel (**A**) reproduced from [34] with permission from Taylor and Francis Group, Informa UK Ltd. www.tandfonline.com (accessed 15 July 2021); Panel (**B**) reproduced from [76,100] with permission from John Wiley and Sons.

FsrC (HK15, EF1820) is the quorum-sensing SHK of the FsrCA two-component system of *E. faecalis* that regulates expression of extracellular proteases gelatinase and serine protease involved in virulence [119,120,121] and biofilm formation [122,123]. Using intact full-length protein, GBAP was shown to stimulate autophosphorylation activity significantly in vitro; two-fold GBAP elicited a 10-fold increase in levels of phosphorylated FsrC (Figure 2) [34]. Pheromone-induced stimulation was confirmed to be specific for FsrC in these in vitro experiments (and not for intact purified SHKs in general), using a range of other purified full-length enterococcal SHK proteins, none of which were activated by GBAP [34]. 

### 3.2. Identification of Direct Inhibitors of SHKs Using Full-Length Intact Proteins: Siamycin I Inhibition of FsrC

**Figure 3:**
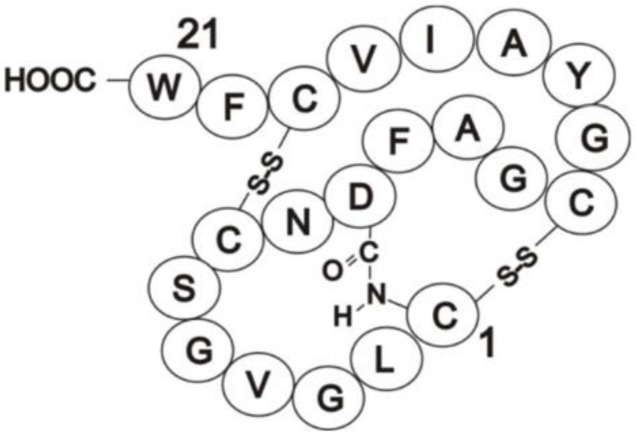
Schematic representation of siamycin I. Reproduced from [124] with permission from the PCCP Owner Societies.

An example of inhibitor identification is provided by siamycin I, a 21-residue tricyclic peptide (Figure 3) [76,124]. Siamycin I was originally identified as an HIV fusion inhibitor, interacting with the HIV envelope protein gp160 [125], and later emerging as an inhibitor of Gram-positive bacteria with minimal inhibitory concentrations ranging from 1.6 to 6.3 µg/mL [126]. Nakayama et al. (2007) first identified siamycin I as an inhibitor of enterococcal Fsr quorum sensing during a natural product screen of actinomycete culture supernatants [84]. They also confirmed growth inhibition of *E. faecalis* (MIC = 2–10 µg/mL) by the peptide inhibitor as well as demonstrating for the first time inhibition of gelatinase production (in the presence or absence of the GBAP pheromone signal), biofilm formation and *fsrBDC* and *gelE-sprE* transcription [84], i.e., quorum sensing. To determine which component of the Fsr quorum pathway was targeted, in particular whether it might be the FsrC membrane SHK (especially as siamycin I disrupts HIV interactions at membrane surfaces [125]), Ma et al. (2011) tested the effect of the inhibitor on the autophosphorylation activity of FsrC [76]. The results demonstrated that FsrC was indeed a direct target for siamycin I (Figure 2; [76]). The peptide inhibitor reduced GBAP-induced autophosphorylation of FsrC by 91%, accounting at least in part for the inhibition of the Fsr pathway activity and gelatinase synthesis reported previously [76]. Inhibition was non-competitive using ATP as substrate and shown to affect other intact SHKs and other ATP-dependent enzymes. 

### 3.3. Binding of the GBAP Pheromone to Purified Intact FsrC

CD spectroscopy was used to determine the strength of GBAP binding to full-length FsrC [74]. Binding was observed in the near-UV region, revealed through changes in the tertiary structural conformation of the protein in the environments of the Tyr and Trp residues. Difference spectra derived from titration experiments in the presence of increasing concentrations of GBAP revealed a k_d_ value of 2 µM for GBAP binding. This demonstrates a moderate, relatively loose binding of GBAP to FsrC, consistent with binding strengths appropriate for a signal transduction protein in which rapid “switch-off” may be as important as activation during fluctuating changes in environmental pheromone levels in vivo. Indeed, the k_d_ value obtained here is similar to that obtained for soluble domains of some other HPKs (e.g., 5.5 µM for the ligand-binding domain of CitA [127]). 

### 3.4. Identification of the Environmental Signalling Ligand That Activates VanS Involved in Regulating Resistance to the Antibiotic Vancomycin

VanS is the membrane-bound SHK component of the VanSR two-component system. Together with its partner RR VanR, it is involved in regulating the *van* genes encoding resistance to the glycopeptide antibiotic vancomycin [128,129]. There are six recognised resistance types (VanA-E, VanG). Bacterial strains carrying VanA-type resistance genes (with a type A VanS, VanS_A_), possess inducible, high-level resistance to both vancomycin (MICs, ≥64 μg/mL) and teicoplanin (MICs, ≥16 μg/mL) glycopeptides [130,131]. VanA-type resistance is induced in vivo by glycopeptides such as vancomycin, teicoplanin, avoparcin or ristocetin, but also by non-glycopeptide agents such as moenomycin, bacitracin, polymyxin B, and robenidine [132], leading to the suggestion that glycopeptides per se are not the primary inducing ligand sensed by VanS_A_. It was suggested that induction may be mediated by the unincorporated Lipid II or early Lipid II precursors that accumulate as a result of the action of all these structurally-diverse cell wall-active agents [133]. However, studies with intact active VanS_A_ revealed no evidence of binding by these precursors in CD spectroscopy experiments [134]. Moreover, it has been suggested that the VanR_A_ (and indeed VanR_B_) is subject to cross-talk by other heterologous SHKs in the absence of VanS, and is fully phosphorylated by these alternative kinases [135,136,137], suggesting that some inducers activate *van* resistance through VanR phosphorylation mediated by alternative SHKs. This provides a plausible explanation for how structurally-diverse inducers can activate VanR.

**Figure 4:**
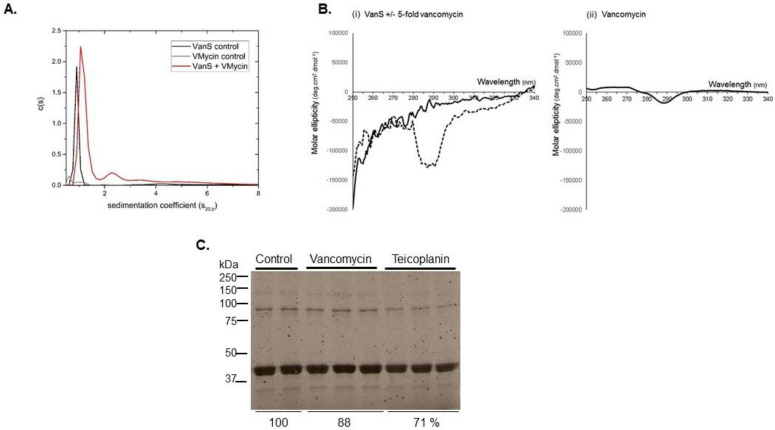
Vancomycin interactions with VanS_A_ revealed through (**A**) analytical ultracentrifugation and (**B**) CD spectroscopy and the effects of glycopeptides on VanS_A_ autophosphorylation (**C**). Panel (**A**): sedimentation coefficient concentration distribution, c(*s*) versus *s* profile for VanS_A_ in HGN buffer (10 mM HEPES pH 7.9, 20% (*v/v*) glycerol, 100 mM NaCl) at 20 °C at a loading concentration of 0.25 mg/mL (5.4 µM). Solubility was greater in buffers lacking added detergent, possibly due to the relatively low hydrophobicity of VanS_A_ with two predicted TMs compared with other membrane proteins. Note that *s* values for the protein are low as the buffer contains 20% glycerol. Black line: VanS_A_; red: VanS + 0.019 mg/mL (12.8 µM) loading concentration of vancomycin; grey: 0.019 mg/mL (12.8 µM) loading concentration of vancomycin (control). Panel (**B**): CD difference spectra obtained in HGN buffer (**i**) solid black line: VanS (9 µM); (**ii**) dashed black line: VanS (9 µM) and vancomycin (45 µM). (**ii**) Vancomycin only control (45 µM). Panel (**C**): Autophosphorylation activity of purified VanS_A_ in the presence and absence of vancomycin and teicoplanin. Purified protein (60 pmoles, 4 µM) was pre-incubated for 20 min at room temperature in the presence of 43 µM vancomycin, 43 µM teicoplanin or buffer solvent, prior to initiation of reactions using 50 µM ATP (containing 3.75 µCi [gamma-^33^P]-ATP). Autophosphorylation was permitted to proceed for 30 min at 22 °C after which time reactions were terminated using stop buffer as described previously [87]. Phosphorylated proteins were separated by SDS-polyacrylamide gel electrophoresis and visualised by autoradiography. Assays were performed in duplicate or triplicate, as shown. Monomeric VanS_A_ is shown at ~42 kDa. % values below lanes are average densitometry values expressed as a percentage of average control values. Panels (**A**,**B**) reproduced from [75] with permission from Springer Nature Ltd.

**Figure 5:**
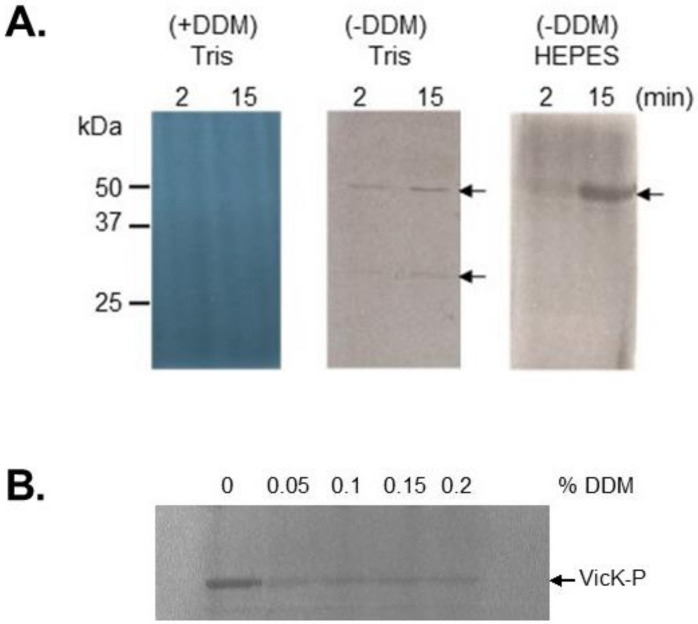
Effect of dodecyl-β-D-maltoside (DDM) and buffer components on autophosphorylation activity of purified enterococcal SHKs EtaS (EF1051) and VicK (EF1194). (**A**) EtaS was purified with 0.05% DDM (+DDM) or without DDM (-DDM) added post-solubilisation in the nickel-NTA purification buffers described previously [34]. The protein (160 pmoles) was then added to reaction buffer (which contains no DDM) [34] containing 10 mM Tris-HCl or HEPES-Na pH 7.6 in a reaction volume of 30 µL and autophosphorylation initiated using radiolabelled ATP as described previously [34]. Samples (15 µL) were removed at 2 and 15 min and reactions terminated. Autoradiographic film of separated proteins are shown. The arrows indicate the positions of phosphorylated EtaS. From [100]; (**B**) VicK was purified in the absence of DDM in the nickel-NTA purification buffers described previously [34]. Autophosphorylation assays using 60 pmol VicK per reaction were undertaken in the presence or absence of a range of DDM concentrations. Samples were removed after 20 min and reactions terminated. Autoradiographic film of separated proteins are shown. The arrow indicates the positions of phosphorylated VicK.

Indeed, vancomycin is able to interact with VanS_A_. This was first demonstrated with binding studies using analytical ultracentrifugation (AUC) and CD spectroscopy studies of the active, folded, full-length VanS_A_ protein and was demonstrated in both the presence or absence of detergents [75] (Figure 4). Binding by teicoplanin was also similarly demonstrated [134]. Both glycopeptides exhibited weak interactions with VanS_A_, exhibiting k_d_ values (~70 µM for vancomycin, 30 and 170 µM for teicoplanin) [134] similar to those obtained for VanS_SC_ binding in in vivo studies [138]. However, there was no resulting stimulation in autophosphorylation activity in response to ~8-fold vancomycin or teicoplanin, even though the purified recombinant protein was well-folded [134] and active [75] (Figure 4). Full-length VanS_A_ was therefore enzymatically insensitive to glycopeptides. The VanS_A_ used in these activity experiments was purified in the presence of DDM detergent but assayed in buffers lacking added detergent to avoid any inhibitory effects of detergents on autophosphorylation activities of some SHKs (e.g., Figure 5). In another study of VanS_A_ in which three different optimised detergents and an amphipol were used throughout the solubilisation and purification steps of protein production and in the activity assays, the same result was obtained; no stimulation in VanS_A_ activities occurred in response to challenge with vancomycin [95]. So regardless of detergent levels, direct interactions between glycopeptide and VanS_A_ can be demonstrated, but these interactions are insufficient to activate the kinase. The most likely explanations are that: (1) vancomycin and teicoplanin do not by themselves serve as the full directly-activating signals for VanS_A_. Since glycopeptides exhibit weak direct interactions with VanS_A_ [75,134], perhaps a signalling complex made up of these glycopeptides together with additional factors serves as the true activating signal for VanS_A_; and/or (2) the presence of the native membrane in which VanS_A_ is embedded is required to obtain VanS_A_ activation in response to direct glycopeptide binding. 

**Figure 6:**
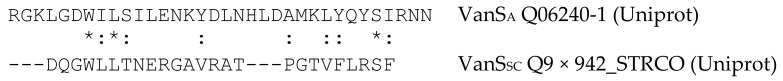
CLUSTAL-O alignment of the predicted extracellular sensory domains of VanS_A_ and VanS_SC_.

Studies of other more distantly-related VanS sensors such as VanS_B_ and VanS_SC_ also revealed direct interactions with vancomycin [94,138], or a signalling complex involving vancomycin [139]. As mentioned earlier, Koteva et al. (2010) reported a k_d_ value of 56 µM for the in vivo binding of a vancomycin photoaffinity probe with actinomycete VanS_SC_ [138], a value comparable to the weak binding strengths observed for VanS_A_ [134], whilst Lockey et al. (2020) demonstrated direct interactions between full-length VanS_SC_ and vancomycin in vitro, and identified regions of the extracellular sensory domain as the interacting site [94]. Inspection of the primary sequence of the sensory domain of VanS_SC_ reveals no significant similarity with VanS_A_ [138,140]; the alignment shown below (Figure 6) is a CLUSTAL-O alignment of the predicted extracellular sensory domains of VanS_A_ and VanS_SC_ (as described in [94]), which share only 8.8% identity.

Therefore, if glycopeptides do indeed serve as the direct activating signal for VanS_A_, the sensing mechanism by which this is achieved is presumably different in VanS_A_ to that in VanS_SC_ (and other unrelated VanS proteins), though operates with similar binding strengths.

### 3.5. Modulation of PrrB Histidine Kinase Activity by the Signalling Cbb_3_-Type Cytochrome C Oxidase Complex in Rhodobacter sphaeroides

The PrrBA two-component system is a global regulator of gene expression in the photosynthetic bacterium *Rhodobacter sphaeroides* [141]. It activates or represses the expression of a large number of genes including photosynthesis, CO_2_ fixation and nitrogen fixation genes (e.g., [141,142,143,144]). Transcriptome analyses revealed that approximately 25% of *R. sphaeroides* genes are regulated by PrrBA, either directly or indirectly [141]. 

PrrB is the membrane-bound SHK component of the PrrBA system and serves as a sensor of changes in redox conditions. It possesses six transmembrane segments and was first expressed as an intact, folded, and active protein by Potter et al. (2002) who demonstrated that PrrB redox sensing did not occur through the direct sensing of oxygen per se, as is the case for some other SHKs such as FixL [87]. Consistent with this, and based on mutant studies, it was suggested that the *cbb_3_*-type cytochrome *c oxidase* complex, which by definition interacts with molecular oxygen, might serve as a regulator of PrrBA activity in response to aerobic/anaerobic conditions [145,146]. Indeed, it was shown that electron flow through the oxidase complex was inversely related to expression levels of photosynthesis genes controlled by PrrBA [147], suggesting that high electron flow through the oxidase might serve as an inhibitory signal that represses PrrBA activity and hence photosynthesis gene expression [147]. Oh et al. (2004) confirmed this in experiments employing purified intact versions of the oxidase and PrrB membrane proteins and PrrA protein, demonstrating that the oxidase complex inhibited activity of intact PrrB in vitro by increasing the intrinsic phosphatase activity of the kinase towards PrrA [92]. The transmembrane domain of PrrB was found to be crucially important for the enhanced PrrB phosphatase activity induced by the oxidase and for optimal autokinase activity suggesting a role for the transmembrane segments in maintaining the correct conformation of the PrrB kinase [92].

### 3.6. Reconstitution of Full-Length SHKs into Phospholipid Liposomes to Study Ligand Interactions: Effects of YycH and YycI Signalling Membrane Proteins on WalK Activities 

An alternative approach for investigating mechanisms of SHK signal sensing and transmission, and for identifying ligands and inhibitors that modulate these processes, is to reconstitute the purified full-length kinases into a membrane-like environment such as phospholipid liposomes [102]. This is particularly appropriate when agonist or antagonist interactions occur within the membrane itself via the transmembrane regions of the kinase. A recent study employing this approach is that of Gajdiss et al. (2020) who investigated the modulatory effects of two accessory membrane proteins, YycH and YycI, on the phosphorylation activities of the *Staphylococcus aureus* WalKR TCS [148]. 

WalKR (also known as YycGF) was first discovered in *Bacillus subtilis* and shown to be essential for growth of the organism [59]. It was shown to regulate essential functions of cell division and cell wall architecture, including *ftsZ* [59,149] and microarray and DNA-binding studies revealed that YycF modulates genes encoding autolysins and autolysin activity [150,151]. Orthologues of YycGF were also shown to be essential for viability in the majority of bacteria investigated, including *S. aureus* [152], in which genes encoding functions related to cell wall metabolism were also found to be under YycGF regulation [153]. YycG was shown to localise to the cell division septum suggesting that signals are perceived at this location in order to regulate autolysin and autolysin inhibitor syntheses and thereby co-ordinate growth and division with cell wall restructuring [154]. This was further confirmed in studies that showed that this translocation process per se did not activate the YycG (WalK) kinase activity, suggesting that the activating signals reside at the division septum destination in the divisome [155]. 

YycG activity is under the regulatory control of two accessory proteins, YycH and YycI, the genes for which are located within the same transcriptional unit as those for the TCS [156,157]. YycH and YycI are membrane proteins that reside in the cytoplasmic membrane and do not locate to the division septum. In *B. subtilis* the combined effects of these two interacting membrane proteins on YycG activity were found to be negative, suggesting a role in suppressing WalK activity when co-located in the cytoplasmic membrane during periods of low growth [155,157]. During periods of active growth and septum formation, WalK locates to the division septum where YycH and YycI are absent. In this location, WalK activity is no longer subject to inhibitory control by these accessory proteins and can become activated by a signal generated by the cell wall biosynthetic complex [155]. 

Interestingly the effects of YycH and YycI on WalK activity in *S. aureus* appear to differ with their counterparts in *B. subtilis*. In *S. aureus*, YycH was found to co-localise with WalK to the division septum and YycH and YycI proteins activated WalK activity, rather than inhibiting it [158,159]. The presence of both accessory proteins together with WalK was required for high level expression of WalKR-expressed genes [158]. One of the aims of the recent study by Gajdiss et al. (2020) of the *S. aureus* system was to obtain direct evidence for the roles of the YycH and YycI proteins in regulation of WalK activity [148]. Using purified full-length versions of all three membrane proteins reconstituted into phospholipid liposomes in various combinations, they showed that YycH and YycI stimulated WalK activity, confirming previous studies, and that both YycH and YycI proteins were necessary for full activation of the kinase in vitro, a finding also confirmed by accompanying in vivo studies [148]. The two opposite regulatory effects of YycH and YycI proteins on WalK kinase activities identified in *S. aureus* and *B. subtilis* and confirmed in this latest study provide an example of how different interaction mechanisms have developed between SHKs and their accessory regulatory proteins amongst different species in order to accommodate their different requirements for controlling and coordinating important cell processes, in this case cell wall remodelling and cell division. 

### 3.7. Reconstitution of Full-Length SHKs into Nanodiscs to Study Ligand Interactions and Signal Transduction: Effects of Metal Ion Ligands on CusS Kinase 

**Figure 7:**
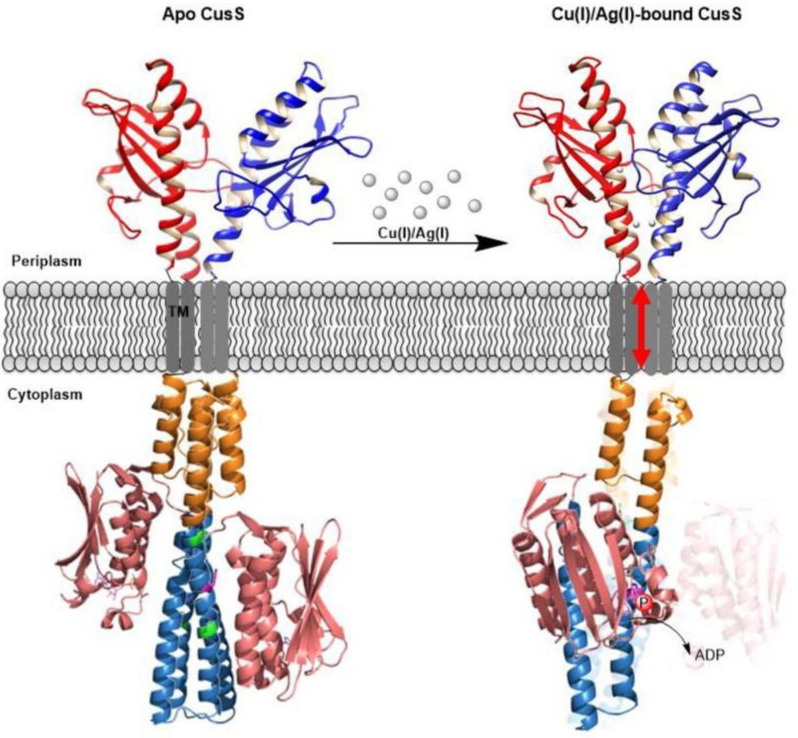
A model for the mechanism of CusS signal sensing and transduction proposed in [109]. In the ligand-unbound apo (inactive) state (leftmost) the sensor domain is asymmetric whilst the cytoplasmic domain is in a symmetric arrangement. In the presence of elevated Ag(I) or Cu(I), the sensor domain dimerises upon metal binding to one of the two binding sites and adopts a symmetric arrangement. This triggers a piston-like movement in the TM domain which in turn causes helical bending in the DHp domain (teal) between the residues shown in green and brings the CA domain (salmon pink) closer to the His271 residue for phosphate transfer. Reproduced from ref. [109] with permission from Portland Press.

A recent study employing nanodisc technology to study full-length SHKs is that of Affandi and McEvoy (2019) [109]. Nanodiscs are soluble nanoscale particles of ~10 nm diameter composed of a lipid bilayer surrounded by a belt or annulus of amphipathic protein known as the membrane scaffold protein, shielding the hydrophobic lipid acyl chains and thereby conferring homogeneity and monodispersity to the particles [104,160]. To prepare nanodiscs containing a membrane protein of interest, detergent-solubilised purified membrane protein is firstly mixed with membrane scaffold protein and lipids (e.g., *E. coli* lipids) in appropriate ratios. Then the detergent is gradually removed whereupon the membrane protein will self-assemble and embed into the nanodiscs which provide a native-like phospholipid bilayer environment, conferring stability, accessibility, and control of membrane protein oligomeric state. Affandi and McEvoy (2019) used a combination of *E. coli* lipids, MSP1D1 scaffold and Empigen BB detergent-solubilised full-length CusS to form nanodiscs which were used to investigate how Cu(I) and Ag(I) ion ligands (natively sensed in the periplasm) activate autophosphorylation in CusS [109]. Having already previously established that only one of the two metal binding sites in CusS was necessary for the crucial dimerization event in the sensory domain, they used the full-length protein in nanodiscs to show that the dimerization event was responsible for activation of the kinase activity in the cytoplasmic domains and they were able to investigate further signal transduction events through to the partner response regulator CusR. Based on these and previous crystal structural data of individual domains they proposed a model for CusS signal transduction based on symmetry-asymmetry transitions, incorporating the effects of metal binding to the one interface binding site in the sensory domain, the resulting dimerization event and kinase activation (Figure 7 ). 

## 4. Extracellular Sensing and Transmembrane Signalling Domains

The majority of SHKs possess extracellular sensing (ESD) and transmembrane (TMD) domains for signal sensing and transmission of signalling information across the membrane. (Although the catalytic cores of SHKs are sometimes referred to as transmitter domains; here, we refer to the terms “signalling” and “transmission” as processes that transfer signals from extracellular sensing domains to the inside of the membrane). ESDs and TMDs are promising potential targets for novel antibiotics, as they are generally well positioned in the cell for drugs that do not need to cross the formidable membrane barrier to be effective. Clearly, structural information on the conformations of these domains in the presence and absence of signalling ligands is important for future drug design strategies aimed at these domains. 

**Figure 8:**
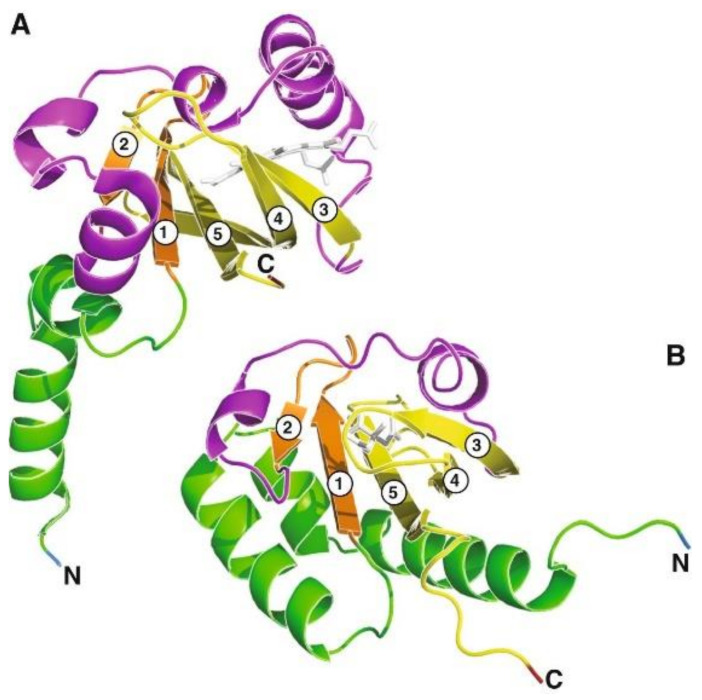
Comparison of the 3-dimensional structures of PAS (**A**,**B**) PDC domains. (**A**) Structure of the PAS domain of *Rhizobium meliloti* oxygen sensor FixL protein with its ligand heme (UniProt P10955: positions 122–251) (PDB: 1D06), and (**B**) Ligand-binding domain of *Klebsiella pneumoniae* CitA protein with its ligand citrate (UniProt P52687: positions 5–135) (PDB: 1P0Z), as described in [161]. Core β strands are labelled from 1 to 5. Colour scheme: the amino end—blue; the leading α-helix region—green; the first two β-strands—orange; the inter-domain α-helix region—magenta; the last three β-strands—yellow; and the carboxyl end—red; ligands—white stick models. Reproduced from ref. [161] with permission from BioMed Central Ltd., Springer Nature.

The structures of ESDs are individually tailored to sense a particular signalling molecule or group of molecules, so in general no consensus conformation is expected amongst different ESDs, which means that drug design aimed at the ESD of one TCS is likely to result in a narrow spectrum drug affecting one process or group of processes in the bacterial cell and likely a limited range of species (thereby potentially targeting individual pathogens with minimal impacts on unrelated beneficial commensals). Many structures of individual ESDs have now been solved, and as more structural information has emerged there are also common themes identified amongst them, including: (i) the PDC domain, a PAS domain possessing different structural arrangements in N- and C-terminal α-helices compared with cytoplasmic PAS domains and named after the first three identified SHKs possessing it—PhoQ, DcuS, and CitA (Figure 8 [161]). Studies with the isolated PAS domain fragment of CitA established that the PAS domain contracts upon citrate binding resulting in a shortening of the C-terminal β-strand [162]. Further studies using liposome-bound protein which comprised all domains apart from the intracellular kinase core domain, demonstrated that this contraction event constitutes the signal transmitted to the TMD [163]; and (ii) the all α-helical sensing domains, which are distinct but topologically similar. Examples include NarX and NarQ SHKs that sense nitrate or nitrite ligands [164,165]; crystallisation of the sensor-TM-HAMP fragment of NarQ in both ligand-free and ligand-bound forms revealed that the sensor domain is a symmetric dimer of two monomers comprising 4 α-helices, with H2 and H4 broken into sub-helices [165]. Whether some of these common regions amongst SHKs can be exploited for inhibitor targeting is currently unknown, but clearly, the more structures that are determined, the more opportunities for exploitation are likely to emerge.

As outlined above, the range of signals sensed by SHKs is very diverse. A recent example of this diversity and accompanying novel and diverse mechanisms of signal perception is provided by HptS [166]. HptS is the histidine kinase component of the HptRSA system involved in responses to glucose-6-phosphate in *S. aureus*. The kinase perceives G-6-P indirectly through the HptA protein which serves as the primary sensor. The mechanism by which glucose-6-phosphate (G-6-P) is sensed by the HptA protein and communicated to HptS was revealed using structural data for the apo and G-6-P-bound states of HptA and for the HptA-HptS complex (using the periplasmic sensing domain of HptS). Whilst the G-6-P-free form of HptA bound to the membrane-distal side of the HptS sensing domain, the G-6-P-bound form switched the contact region with HptS to the membrane-proximal domain, in an interface switch-mediated form of signal transduction [166]. 

Whilst signals and sensing domains generally exhibit diversity, the question arises as to whether signal transmission to the TMs is also diverse, or whether there are conserved regions/processes common to many SHKs that are potentially more amenable for broad spectrum drug design. The sensing domains of many SHKs possess an extended helix (the p(eriplasmic)-helix) at the dimer interface that lies proximally to the ligand binding site, which connects to the TMs [167]. It has been suggested that ligand binding causes a conformational change in this helix which in turn affects the conformation of the TMs [167]. Several models for signal transmission have been proposed, including (a) the asymmetric piston shift model, in which one of the two p-helices in the dimer moves into the cytoplasm; (b) diagonal scissoring of the two p-helices; and (c) helical rotation of the two p-helices (reviewed in [9]). These mechanisms are not mutually exclusive. Indeed, all three mechanisms were recently shown to occur during signal transmission in *E. coli* NarQ [168]. Using crystal data derived from a sensor-TM-HAMP fragment locked in a ligand-bound-like conformation, and in its ligand-free conformation, molecular dynamic simulations revealed that binding by the nitrate ligand firstly caused helical rotation and diagonal scissoring of the alpha helices at the centre of the sensory domain. These changes were also accompanied by a small piston-like motion which was amplified by a switch in the conformation of the linker located between the sensory and TM domains [168]. Other combinations of more than one of these mechanisms have been observed previously (see [9]) and Bhate et al. (2015) proposed that a common theme amongst several reports of transitions from apo- to ligand-bound states is a transition between symmetric and asymmetric states [167]. 

The majority of SHKs possess two transmembrane helices per monomer and dimerise in vivo, suggesting that TMDs form a four-helix bundle across the membrane which provides the means by which signalling information is transduced across the membrane and into the cytoplasm [167]. Some SHKs that have more than two transmembrane segments, including FsrC and PrrB described above (both of which possess six predicted TMs per monomer) and DesK (5 TMs per monomer). Regions of hydrophilicity within (or close to) TMDs are proving to be important for signal sensing and transduction in some SHKs. For instance, in the case of DesK, which senses changes in temperature via the thickness of the membrane, experiments using reduced numbers of TMs showed that just one chimeric TM (composed of parts of TMs 1 and 5) was sufficient for thermal sensing and that just three hydrophilic polar residues (Q9, K10, and N12) located within a hydrophilic “hotspot” near the lipid-water interface in the TM were essential [169]. The authors suggested that membrane thickness controls the signalling state of DesK by affecting the hydration level of this hydrophilic region. Fernández et al. (2019) recently showed that membrane fluidity signals are then transmitted to effector domains via rotation of a connecting 2-helix coiled coil domain, and that more hydrophobic TM proline residues were important for signalling to this 2-HCC [170]. The importance of hydrophilic residues within or close to TMDs for transmembrane signal transfer has been identified in other studies too, as highlighted in [9]. For example, a polar residue located centrally within the TMD of PhoQ was suggested to form a water pocket and shown to be important for signal transduction [171,172], whilst interactions between signalling protein WalI and the WalK SHK within the membrane are mediated by hydrophilic residues [157,173]. 

## 5. Conclusions and Perspectives

In this review we have highlighted some of the recent advances made in knowledge of the structure and function of membrane SHK signal sensing and transmembrane signal transduction in studies that have employed intact versions and domain fragments of these proteins involved in sensing and early signalling events. These portions of membrane SHKs are commonly located extracellularly and within the cytoplasmic membrane, locations that may be suitable for novel antibacterial drugs that are not able to cross the membrane barrier. Even though the range of signalling ligands sensed by SHKs is diverse, and there are a range of different sensing and signalling mechanisms in these proteins, previous and recent structural studies indicate the presence of a small number of regions (e.g., PDC, all α-helical domains and p-helices) and functions that are shared amongst some SHKs; it is currently uncertain whether these are sufficiently large or distinctive to feasibly be exploited in drug discovery strategies, especially as some are shared by other membrane receptors. 

The use of full-length versions of these membrane proteins in signalling studies has been increasing in recent years. Full-length proteins possess their native ligand/signal binding sites linked to their native assayable phosphorylation functions and so whilst there are clearly more technical challenges associated with purifying the full-length versions of these membrane proteins rather than their soluble counterparts lacking their transmembrane domains, there are also distinct advantages for doing so:

(i) They lend themselves particularly well to investigations aimed at identification of environmental signals together with characterisation of their downstream impacts on enzyme activities and structural conformations, especially across the membrane. In this review, we have described the advances in knowledge of ligand binding by CusS, FsrC, PrrB, VanS_A_, VanS_SC_, and WalK and their effects on activities and/or mechanisms of signal transmission. This should apply equally well to identification and characterisation of inhibitors; one study using intact FsrC successfully identified siamycin I as a direct inhibitor and therefore it should be possible to expand this approach. Quantitative data for ligand binding in the presence of the entire intact proteins is also achievable, though from the few studies to date it seems that ligand binding strengths are generally comparable to those obtained using sensing domains in isolation which are often more convenient to produce; and
(ii) Intact membrane SHKs can be used to investigate ligand interactions that occur within the membrane itself. Indeed, the first description of the successful heterologous expression and purification of any intact membrane SHK came from studies of an intramembranous-sensing SHK—PrrB (RegB) from Rhodobacter sphaeroides. Intact protein studies established that the membrane-bound cbb_3_-type cytochrome *c oxidase* complex serves as the interacting regulator of PrrB kinase/phosphatase activities and that the PrrB transmembrane domain plays a crucial role in the interactions.

The above examples are studies of individual signals and individual SHK proteins. When it comes to using intact membrane SHKs in rapid high throughput screening to identify new inhibitors for development as new antibiotics, significant challenges arise because the analytical techniques available to identify inhibitor binding or inhibition of activity are not suitable for high throughput screening formats. However, intact SHKs might be usefully incorporated within adapted activity-based screens that currently employ domain fragments, which would broaden the number of potential target sites tested simultaneously within one test. 

Another important area in which intact SHKs could contribute significantly to antibiotic discovery in the future is elucidation of the three-dimensional structures of full-length, membrane-bound SHKs in the presence and absence of ligands (and indeed inhibitors). To the best of our knowledge, no such structures, either in the presence or absence of membranes, have yet been reported and therefore a complete understanding of common themes of transmembrane signal transmission and links to downstream events from multiple structures is still lacking. Recent advances in cryo-electron microscopy have yielded high resolution structures of an increasing number of membrane proteins. Since SHKs are highly flexible, rendering crystallisation approaches less viable, structural determinations by cryo-EM may be a suitable way forward. There have also been recent advances in methods for obtaining solubilised intact membrane proteins without use of detergents (or reduced detergent) which can be inhibitory for or destabilise some SHKs. These methods (amphipols, liposomes and nanodisc and other lipid/polymer encapsulation technologies), have already been successfully applied to some studies of membrane SHKs and offer increased possibilities of producing more stable full-length proteins for crystallisation in the future. Perhaps advances such as these, together with other developments described in this review might encourage more researchers to explore the possibilities using full-length SHKs in the future. 

## Figures and Tables

**Table 1:** Numbers of known or predicted two-component signal transduction systems in examples of pathogenic bacteria, and correlation with genome size.

Species	Genome Size (Mb)	SHKs *	RRs *	Reference
*Burkholderia pseudomallei* NCTC13179	7.24	42 + 7 **	62	[35]; http://www.p2cs.org (accessed on 1 August 2021)
*Pseudomonas aeruginosa* PAO1	6.26	53	83	[15,36]
*Bacillus anthracis* Ames Ancestor	5.50	47	47	[37]; http://www.p2cs.org (accessed on 1 August 2021)
*Escherichia coli* K12 W3110	4.64	30	34	[32,38]
*Mycobacterium tuberculosis* H37Rv	4.41	16	13	[39]; http://www.p2cs.org (accessed on 1 August 2021)
*Bordetella pertussis* Tohama I	4.09	19	22	[40]; http://www.p2cs.org (accessed on 1 August 2021)
*Acinetobacter baumanii* ATCC17978	3.98	19	19	[41,42]
*Enterococcus faecalis* V583	3.36	17	18	[33,34,43]
*Brucella melitensis* 16M	3.29	22	24	[44]
*Brucella abortus* 9-941	3.28	22	24	[45]
*Clostridium perfringens* 13	3.03	28	20	[46]
*Staphylococcus aureus* Mu50 (methicillin- and vancomycin-resistant)	2.90	17	17	[47]
*Staphylococcus aureus* N315 (methicillin-resistant)	2.84	17	17	[47]
*Streptococcus agalactiae serogroup III strain NEM316*	2.21	21	22	[48,49]
*Streptococcus pneumoniae* 19F	2.10	13	13	[50,51]
*Streptococcus pyogenes* M1	1.85	13	13	[52]
*Haemophilus influenzae* Rd KW20	1.83	3	6	[53]; http://www.p2cs.org (accessed on 1 August 2021)
*Helicobacter pylori* 26695	1.67	4	7	[54]
*Campylobacter jejuni subsp. jejuni* NCTC 11168	1.64	7	12	[55]; http://www.p2cs.org (accessed on 1 August 2021)
*Mycoplasma genitalium* ATCC 33530	0.58	0	0	[56]

* includes any hybrid sensor-regulators unless otherwise denoted; for example, ** (+) values denote number of hybrid sensor-regulators. http://www.p2cs.org (accessed on 16 September 2020).

**Table 2:** Rates of success in each step during the heterologous expression and purification of active intact membrane SHKs of *Enterococcus faecalis*. From Ma et al. (2008) [34].

	Success Rate * (Number)	Success Rate * (%)
Expression in *E. coli*	15	94
Active when assayed in *E. coli* membranes ^†^	11	73
Detergent solubilisation	14	93
Purification (using hexa-His tag)	13	87
Purification and confirmation of intact protein	12	80
Purified as active protein ^†^	13	87

* For expression, values are out of a total of 16 membrane SHKs; for other steps, values are out of a total of 15 (i.e., of those expressed). ^†^ Autophosphorylation activity in the absence of signalling ligand.
